# Core harm outcome sets developed by drug are needed to allow informed risk-benefit decision making

**DOI:** 10.1186/1745-6215-16-S2-P55

**Published:** 2015-11-16

**Authors:** Victoria Cornelius, Kun Liu, Janet Peacock, Odile Sauzet

**Affiliations:** 1 King's College London, London, UK; 2 University of Bielefeld, Bielefeld, Germany

## 

Good information on drug harm is vital to inform risk-benefit decisions and undertake robust cost effectiveness analysis. Harm information in peer-reviewed articles have been found not to be useful for this purpose. Regulators require pharmaceutical companies to produce product information documents (Europe:SmPC, US:USPI). These documents contain comprehensive information on the known harm of a drug. We reviewed the usefulness of the data and compared the harm profile for brand drugs marketed in Europe and the US. This review included 12 antidepressants/antiepileptic brand drugs (SmPC and USPI).

On average 77 more adverse reactions (ARs) were reported in the USPI compared to the SmPC document. The median number of ARs included were SPC:114 (IQR 93,150) and USPI:201 (IQR 114, 262). In clinical trials many adverse events (AEs) will be reported for each treatment. In order to identify ARs from all the AEs reported during the trial, a criterion or rule is often used. Nine USPIs and 3 SmPC reported the criterion used, but this was not the same in the 3 paired documents and in 7 USPIs multiple criterion were used.

It is expected that the harm profile in the product information for the same drug should agree. This review found a lack of consistency for the same drug and demonstrates the overwhelming impact of arbitrary rules used for reporting. This problem can only be exacerbated across drugs.

The development of CORE harm outcome sets by drug class would facilitate comparison of harm profiles across drug and support informed risk-benefit decisions.

